# Network enrichment analysis: extension of gene-set enrichment analysis to gene networks

**DOI:** 10.1186/1471-2105-13-226

**Published:** 2012-09-11

**Authors:** Andrey Alexeyenko, Woojoo Lee, Maria Pernemalm, Justin Guegan, Philippe Dessen, Vladimir Lazar, Janne Lehtiö, Yudi Pawitan

**Affiliations:** 1 School of Biotechnology, Royal Institute of Technology, Stockholm, Sweden; 2, Science for Life Laboratory, Stockholm, Sweden; 3 Department of Statistics, Inha University, Incheon, South Korea; 4 Clinical Proteomics Mass Spectrometry, Karolinska Institutet, Stockholm, Sweden; 5 Functional Genomics, Institut Gustave Roussy, Villejuif, France; 6 Department of Medical Epidemiology and Biostatistics, Karolinska Institutet, Stockholm, Sweden

## Abstract

**Background:**

Gene-set enrichment analyses (GEA or GSEA) are commonly used for biological characterization of an experimental gene-set. This is done by finding known functional categories, such as pathways or Gene Ontology terms, that are over-represented in the experimental set; the assessment is based on an overlap statistic. Rich biological information in terms of gene interaction network is now widely available, but this topological information is not used by GEA, so there is a need for methods that exploit this type of information in high-throughput data analysis.

**Results:**

We developed a method of network enrichment analysis (NEA) that extends the overlap statistic in GEA to network links between genes in the experimental set and those in the functional categories. For the crucial step in statistical inference, we developed a fast network randomization algorithm in order to obtain the distribution of any network statistic under the null hypothesis of no association between an experimental gene-set and a functional category. We illustrate the NEA method using gene and protein expression data from a lung cancer study.

**Conclusions:**

The results indicate that the NEA method is more powerful than the traditional GEA, primarily because the relationships between gene sets were more strongly captured by network connectivity rather than by simple overlaps.

## Background

Gene-set enrichment analyses (GEA or GSEA) [1-3] are commonly used to characterize experimentally derived altered gene sets (AGSs). The ‘alteration’ is associated with a certain biological state, such as a cell culture perturbation or a malignant tumor. (Later we will distinguish GEA from GSEA, but to avoid repetitiveness in this Introduction we will use GEA as a generic concept. Since there are many frequently-used acronyms in this paper, we tabulate them in Table 1.) A typical AGS is a list of differentially expressed (DE) genes, and GEA identifies which previously known functional gene sets (FGSs) are over-represented in AGSs. Although this is both informative and intuitively clear, the majority of genes have not been assigned to any biologically informative category, i.e. a Gene Ontology (GO) term or a pathway. The sensitivity of these analyses is limited by the AGS size, but expanding the set to include low-ranked genes (e.g. less differentially expressed) is not always biologically meaningful. Moreover, the experimental platform suggesting the AGS (e.g. gene expression array) might be biologically disjoint from the major mechanisms of activity in the FGS (e.g., mutation or protein phosphorylation).

**Table 1:** List of acronyms and their meanings

**Acronym**	**Full word**	**Brief description**
AGS	Altered gene set	A list of differentially expressed genes in experiment
FGS	Functional gene set	A list of genes that were previously known to be related
GEA	Gene enrichment analysis	A method of counting overlaps between AGS and FGS
GSEA	Gene set enrichment analysis	A method using the maximum overlap between AGS and FGS
NEA	Network enrichment analysis	A method of measuring network connectivity between AGS and FGS
FNEA	Fixed network enrichment analysis	A NEA method when AGS is a list of *k* top-ranking genes
MNEA	Maximum network enrichment analysis	A NEA method to avoid dependence on *k*

In parallel with the development of GEA and expression measurement technology, biological knowledge about gene and protein interaction is also growing rapidly. Gene networks readily connect well- and poorly annotated proteins, and combine various biological mechanisms. A few examples of current applications include: inference concerning the functions of previously unannotated genes [4], extension of GO terms and pathways [5], finding novel disease genes [6] and prioritization of disease gene candidates [7].

There are currently a number of network-based methods that characterize gene sets rather than individual genes. Similarly to GEA, some methods can be considered network enrichment analysis (NEA). Such approaches have revealed network patterns that are enriched compared to those expected by chance. For example, using an information-theoretic method, Huttenhower *et al.*[8] analytically derived scores to quantify network relations between distinct biological processes from GO terms. Shojaie and Michailidis [9] suggested a linear model approach within an orthogonal microarray design and employed both the gene expression data and a previously known gene network to detect GO terms most influential in the transcriptome perturbation. Unfortunately, this method would require extensive modification and statistical analyses for every new application.

The existing network-based methods have not fully integrated the topological information in the gene network and the functional information about biological processes. JActiveModules [10] is a popular and easy to use software, as it is run on the state-of-the-art network browser platform Cytoscape [11]. The idea is to find modules in the global network based on the significance of gene alteration (usually differential-expression p-values). Note here that these modules are not biologically characterized by any known FGS. A similar method was suggested in Liu at al. [12], using their own software. As in JActiveModules, network modules enriched in DE genes are first identified, and these modules serve as the AGSs. The biological characterization of the identified modules proceeds in the same way as GEA: known FGSs are assessed for being over-represented in the AGSs using Fisher’s exact tests. Hence, the network topology influences the formation of AGS, but not the relationships between AGS and FGS. Another similar approach was implemented in the Ingenuity Pathway Analysis software (IPA; http://www.ingenuity.com). Lists of differentially expressed [13] or alternatively spliced genes [14] were found to be associated with certain biological processes within limited ‘gene networks’ (using IPA terminology). To our knowledge, none of the methods developed so far offers a straightforward and transparent interpretation comparable to GEA. Such a method should exploit the topological properties of the networks, in particular the interactions between the AGS and FGS, and should provide a proper statistical inference.

In this paper we present a method of network enrichment analysis that systematically implements the network approach to describe novel gene sets with biologically meaningful functional categories. The method integrates two kinds of biological information: functional information (currently available for a minority of genes) and network connectivity of nearly all protein-coding genes. In contrast to traditional GEA, our NEA method quantifies the over/under-representation of the functional group members among the neighbors in the gene network rather than in the AGS itself. For statistical inference, we developed a fast network randomization algorithm in order to obtain the distribution of any network statistic under the null hypothesis of no association between an AGS and FGS. We demonstrate applications of NEA on high-throughput gene and protein expression datasets from a lung cancer study, and compare its performance and power with those of GEA. We provide an R package called ‘nea’, freely available at http://http:/www.meb.ki.se/~yudpaw. For pedagogic purposes, in Section C of the Additional file 1, we also provide a fully worked-out example of analysis of lung cancer data from Bild *et al.*’s [15].

## Methods

### Data from a lung cancer study

For illustration we used expression data from a cohort of 123 lung cancer patients who underwent complete surgical resection at the Institut Mutualiste Montsouris (Paris, France) between 30 January 2002 and 26 June 2006. The recruitment of participants was carried out in compliance with the Helsinki Declaration, and this study was approved by the ethics committee of the Institut Gustave Roussy (Paris). All involved patients gave a written informed consent. From each patient, snap-frozen tumor and corresponding normal tissues were collected. We assayed the samples for gene expression, performed using dual-color human array from Agilent containing 41,000 gene probes; a dye-swap was employed for each sample and the log-ratio value was combined by averaging. Scanned microarray images were analyzed by using Agilent Feature Extraction software version 10.5.1.1. A loess normalization was then performed using the normalizeWithinArrays function from R package limma [16].

For a subset of 16 tumor samples we also obtained protein expression profiles. From each sample 160 *μ*g of protein was taken off and precipitated using four volumes of ice cold acetone. The precipitated samples were dissolved in iTRAQ dissolution buffer and digested according to manufacturers instructions (Applied Biosystems). Three proteomics platforms were used: Applied Biosystems’s MALDI TOF-TOF, Agilent’s Accurate-Mass Q-TOF and a hybrid LTQ-Orbitrap from Thermo Fischer Scientific. From these platforms, a total of 4,406 proteins were detected in at least one sample.

### Altered gene sets

AGSs are usually defined as genes that are differentially expressed (DE) between clinical groups. In our current application we defined the AGS as the DE genes in the within-person comparison of tumor vs normal tissues, so each sample (patient) had an associated AGS. The top-ranking genes are not interpreted statistically significant DE genes, but as potentially deregulated genes. To investigate the functional heterogeneity of individual tumors, we ranked the differences between individuals. To this end, we transformed the mRNA expression data to difference values that conveyed how uncommon is the differential expression of gene *g* in patient *p* compared to the group of patients:

(1) $$\log{\left(T/N\right)}_{\mathrm{pg}}-\text{ave}{\left(\log(T/N)\right)}_{\mathrm{.g}},$$

where log(*T*/*N*) is the log intensity-ratio of tumor vs normal expression. For the AGS with a fixed size, we considered the top 100 (*k* = 100) genes per individual.

The AGS from the protein expression data was defined similarly: for each sample we kept proteins whose expression exceeded the corresponding average across 16 samples. The number of proteins in the AGSs has a median of 424 (range 257 to 654).

### NEA statistics and randomization

Let *A*(*k*) be an AGS of size *k*, and define *n*_*AF*_(*k*), a measure of network connectivity between *A*(*k*) and a known FGS (*F*), as the number of links between members of *A*(*k*) and *F*. In practice we might be interested to see the network connectivity for a fixed number *k* of top-ranking genes. Since the connectivity depends on the constituent genes, we correct *n*_*AF*_(*k*) by its expected value:

$$d_{\mathrm{AF}}(k)=n_{\mathrm{AF}}(k)-\mu_{\mathrm{AF}}(k)$$

where *μ*_*AF*_(*k*) is the expected number of links between *A*(*k*) and *F*. The computation of *μ*_*AF*_(*k*) is performed using the network randomization described below. To avoid dependence on *k*, we define the largest (signed) deviation between *n*_*AF*_(*k*) and its expected value:

$$\begin{array}{lll} d_{\max}\; & =\;\underset{k}{\max}\{n_{\mathrm{AF}}(k)-\mu_{\mathrm{AF}}(k)\}\; & \;\\ d_{\min}\; & =\;\underset{k}{\min}\{n_{\mathrm{AF}}(k)-\mu_{\mathrm{AF}}(k)\}\; & \;\\ d_{\mathrm{AF}}\; & =\;\left\{\begin{array}{cc} d_{\max},\; & \mathrm{if}\;d_{\max}\geq -d_{\min}\\ d_{\min},\; & \mathrm{if}\;d_{\min}\leq -d_{\max}. \end{array}\right)\; & \; \end{array}$$

The immediate statistical question is whether an observed *d*_*AF*_(*k*) or *d*_*AF*_ is significantly higher or lower than expected under the null hypothesis of no biological relation. This assessment is not trivial, as we need to respect the network topology. Crucially, it is not adequate to compare the AGS with a random list of genes. The scale-free property of most biological networks means that the degree distribution is highly skewed, with a few network hubs but a large number of sparsely connected nodes. (The degree of a node is defined as the number of neighbors connected to that node). Randomly replacing a network hub by a sparsely connected gene does not make sense. A hub gene may have a few links to genes in a FGS simply by chance, whereas for a non-hub gene the same number of links would indicate an important biological pattern.

In some simple cases, analytical calculations of the null distribution are possible, for example using the hyper-geometric distribution [17]. However, for general networks, e.g. when gene groups may share members, theoretical analyses are not feasible. In such a situation, for example, Li *et al.*[18] chose to limit their analysis to only cases where the gene groups did not overlap, which is a serious limitation.

A general approach in generating the null distribution of the network statistic is based on a network randomization that preserved the degree distribution. This approach was applied, for example, by [19-22]. The basic algorithm rewires the whole network by systematic swapping of network links so that the degree of each node is preserved, while its network neighbors are replaced. The algorithm is as follows: 

Step 0: Randomly select a pair of edges (A-B and C-D).

Step 1: ‘Rewire’ the two edges in such a way that A becomes connected to D, while B connects to C. In case one or both of these new links already exist in the network, this step is aborted and a new pair of edges is selected.

Repeated applications of the above steps lead to a randomized version of the original networks. However, for a large network it is not clear at what point a sufficient level of rewiring has been achieved, and the algorithm is also inefficient as it rejects too many rewired links that are generated earlier.

The key to a fast and transparent algorithm is to first represent the network in terms of a binary adjacency table, with each node represented by a row and a column, and the cell entry ‘1’ represents connected nodes, and ‘0’ otherwise. The table is symmetric, and its margin is equal to the degree distribution of the nodes. Thus our goal is to generate a randomized table with fixed margins, similar to the hypergeometric sampling model associated with the Fisher’s exact test, but with the additional constraints of symmetry and binary entries. Adapting the hypergeometric sampling model to these constraints, the network randomization is achieved by the following sequential algorithm: 

Step 0: Start with a list of nodes 1,…,*N*, ordered by degree *δ*_1_ ≥ ⋯ ≥ *δ*_*N*_> 0.

Step 1: Assign the *δ*_1_ links of the largest node to the other nodes randomly but with probability weighted by *δ*_2_, …, *δ*_*N*_.

Step 2: Remove node 1, and reduce by 1 the degree of each node connected to node 1.

Step 3: Construct a new list of remaining nodes with positive degrees, then reorder and renumber them 1,…,*N*, and go to Step 1. Stop if there is no remaining node with positive degree.

At first appearance the use of the weights ( *δ*_2_,⋯, *δ*_*N*_) makes it more difficult to understand the distributional property of generated networks. Here we explain why the weights lead to an unbiased random sample of networks with a given degree distribution. First consider a naive algorithm for generating networks uniformly from all networks with the given degree distribution: 

1.Construct a random network: for *i* = 1,⋯,*N*, take *δ*_*i*_ nodes randomly with equal weights as neighbors of the *i*th node. The choices of neighbors are made independently across different nodes.

2.Test the network: compute the resulting degree distribution and accept the network if it has the required degree distribution, otherwise reject it.

Since a great majority of generated networks will not satisfy the given degree distribution, and will thus be rejected, this algorithm is highly inefficient. However, clearly the accepted/generated networks are uniformly distributed over all networks with the given degree distribution. Now, at Step 1 the probability that the second largest node has a link with the largest node is

$$\begin{array}{ll} \frac{\left(\begin{array}{c} N-2\\ \delta_{2}-1 \end{array}\right)}{\left(\begin{array}{c} N-1\\ \;\;\delta_{2} \end{array}\right)}=\frac{\delta_{2}}{N-1} & \; \end{array}$$

Likewise, the probability that the node with degree *δ*_*i*_ has a link with the largest node is $\frac{\delta_{i}}{N-1}$. This is exactly the same as the weights in our algorithm. Thus, our weighting scheme simply offers a short-cut to produce truly unbiased random networks. A similar proof was used in [23]. Our proposed algorithm is computationally very efficient, since each node needs to be visited at most once. The average number of nodes that need to be visited depends on the degree distribution. In our application with ∼16,000 nodes and ∼1.5 M links (see the subsection on gene network below), this average was ∼8,200 nodes. On a 3 GhZ PC with 4 Gb RAM, using native R commands, the algorithm takes ∼60 seconds.

**Figure 1:**
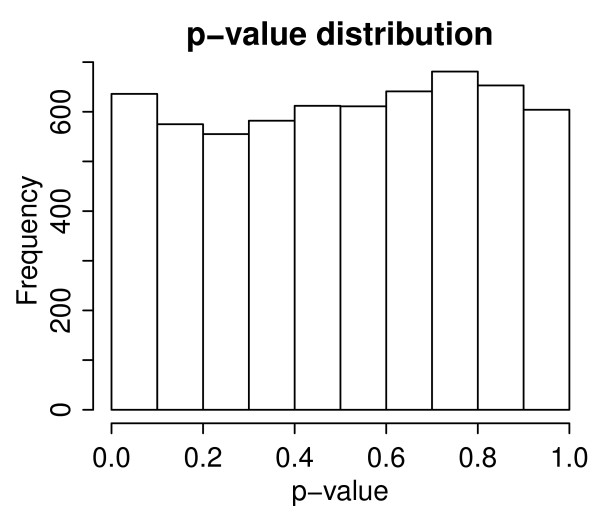
**The distribution of the p-values of the statistics computed from the randomized network.** If the randomization works well, then the distribution should be uniform.

Denote by $n_{\mathrm{AF}}^{*}(k)$ the number of links between *A*(*k*) and *F* based on the randomized network. On replicating the randomization many times (in practice we used 100 replications), we obtain of collection of $n_{\mathrm{AF}}^{*}(k)$’s, which behave as a random sample from the null distribution. We can then estimate *μ*_*AF*_(*k*) by the average of $n_{\mathrm{AF}}^{*}(k)$ over the randomizations, and compute $d_{\mathrm{AF}}^{*}(k)$ and $d_{\mathrm{AF}}^{*}$ based on the randomized network, and compute the right-sided p-value as the proportion of $d_{\mathrm{AF}}^{*}$’s ≥ *d*_*AF*_(similarly the left-sided p-value). The one-sided p-value is the minimum of these two, and the two-sided p-value is twice the one-sided p-value. We checked that the p-values computed for $d_{\mathrm{AF}}^{*}$ for a typical FGS (with median size), across all AGSs and all the randomized networks, followed a uniform distribution, indicating that the randomization worked as expected; see Figure 1.

For easy comparison with other methods we computed the maximum NEA (MNEA) score as

$$z=\frac{d_{\mathrm{AF}}-{\bar{d}}_{\mathrm{AF}}}{s_{\mathrm{AF}}},$$

where ${\bar{d}}_{\mathrm{AF}}$ and *s*_*AF*_ are the mean and standard deviation of $d_{\mathrm{AF}}^{*}$. Similarly, the fixed NEA (FNEA) score is computed as

$$z(k)=\frac{d_{\mathrm{AF}}(k)-{\bar{d}}_{\mathrm{AF}}(k)}{s_{\mathrm{AF}}(k)},$$

where ${\bar{d}}_{\mathrm{AF}}(k)$ and _*sAF*_(*k*) are the mean and standard deviation of $d_{\mathrm{AF}}^{*}(k)$. We use the term NEA when referring to both FNEA and MNEA. These scores can be used as a measure of activation of the FGS, thus providing a biological characterization of the AGS. Assuming a normal distribution, one could convert the scores to p-values, which are convenient for combining results of network analyses with information from other findings, such as genome-wide association studies, small-scale experiments, mutation analysis, etc.

To assess a large number of AGS-FGS pairs, we transform the p-values into false discovery rates (FDR) [24]. Let *p*_1_,…, *p*_*m*_ be the ordered p-values from *m* AGS-FGS pairs. The standard estimate of FDR as a function of the p-values is given by

$$\text{FDR}(p_{k})=m{\hat{\Pi }}_{0}p_{k}/k,$$

where ${\hat{\Pi }}_{0}$ is the estimated proportion of null results. Monotonicity is then imposed by taking the cumulative minimum over all p-values ≥ *p*_*k*_.

### Comparative procedures: GEA and GSEA

For comparisons with NEA, the GEA score was computed from an AGS of size *k* as follows. Firstly, a standard 2×2 table is constructed for each AGS-FGS pair according to whether or not a gene belongs to the AGS and FGS. In small samples the Fisher’s exact p-value is usually computed using the hyper-geometric distribution, which, as explained above, is equivalent to randomizing the genes while fixing the marginal totals. Liu *et al.*[12] used this assessment for their statistic, which is why the method was more like a GEA rather than a true NEA, as it ignored the network topology in its inference. For convenience and easy comparison with NEA scores, we compute a GEA score as

$$z_{k}=\frac{\log(\mathrm{ad}/\mathrm{bc})}{{\left(1/a+1/b+1/c+1/d\right)}^{1/2}},$$

where (*abcd*) are the table frequencies. This can be seen as the *z*-statistic associated with the log odds-ratio. If some observations in the table are zero, then they are replaced with 0.5 to prevent *z* from being undefined.

Similar to MNEA, the gene-set enrichment analysis (GSEA) avoids the dependence on *k* by taking the maximum deviation from zero encountered in a running sum statistic [1]. GSEA enrichment score is defined as

$$\begin{array}{ll} \underset{i}{\max}\left|\underset{g_{j}\in \text{FGS},j\leq i}{\sum }\frac{1}{N_{\text{AGS}\cap \text{FGS}}}-\underset{g_{j}\notin \text{FGS},j\leq i}{\sum }\frac{1}{N_{\text{AGS}}-N_{\text{FGS}}}\right|, & \; \end{array}$$

where *g*_*j*_ is the *j*th-ranked gene in the AGS, *N*_*R*_is the number of genes in *R*. As with the GEA, however, GSEA also does not use any network topology in its inference. To assess the significance of observed enrichment score, a permutation test procedure was originally developed for group comparisons. In our application the AGS is defined for each person, so the permutation is performed within each person.

### Gene network

We compiled a comprehensive network by merging 4 networks: (1) the data integration network of functional coupling [21], re-generated with updated datasets on protein-protein interactions (IntAct), gene expression (GEO) and sub-cellular localization (GO). This network contains 1,515,318 links, with each gene identified by the ENSEMBL gene ID; (2) curated pathways (KEGG KGML files, as of June 2010), consisting of 25,765 links; (3) known protein complexes (KEGG and CORUM, as of June 2010), consisting of 54,046 links; (4) coherent annotations (coherence was estimated by a custom software as simultaneous presence of two genes in a series of overlapping GO terms, weighted for compactness of the latter), consisting of 20,412 links.

The final network contained 1,445,027 functional links between 16,299 distinct HUPO genes (some links were lost as we translated the ENSEMBL IDs to HUPO gene symbols). Note that no evidence from the lung cancer datasets was involved in the network construction. The network is available from http://www.meb.ki.se/~yudpaw.

### Functional gene sets

To characterize AGSs by involvement of known biological processes, we compiled a list of genes which are of importance in cancer: 

All 235 pathways in the KEGG database (as of 21 Apr. 2010);

16 GO terms that could be related to hallmarks of cancer [25];

7 cancer-related pathways and otherwise defined FGS from publications reporting on large-scale cancer genome projects;

EMT, epithelial-mesenchymal transition (a pathway behind metastatic development, manual curation by S. Souchelnytskyi);

Acidic switch (a pathway behind tumor-specific pH-shift, i.e. tumor’s ability to grow in hypoxic environment, manual curation by A. de Milito).

In total the list included 5,698 distinct HUPO gene symbols assigned to 264 FGSs (overlaps are allowed). The list of pathways with their meanings and references are given in Section B of the Additional file 1 Report. The complete list of genes for each FGS is available as an RData file at http://http:/www.meb.ki.se/~yudpaw.

## Result and discussion

### Simple illustration

**Figure 2:**
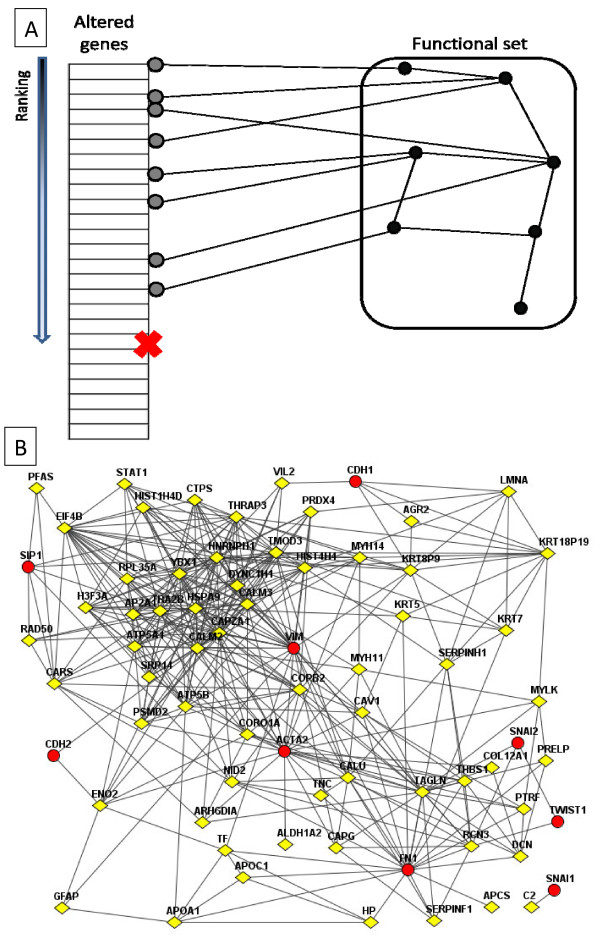
**A schematic diagram of counting network links and a real example of network links.(A)** A simple example of how links are counted between genes in AGS and FGS. The ’x’ symbol indicates a fixed number *k* of genes; **(B)** A realistic example of network links between 257 deregulated proteins in a tumor (diamonds) and 10 genes known to be involved in the epithelial-mesenchymal transition (circles). Network nodes without links to the AGS or FGS genes are not shown. The graph is generated using a graphics tool in the FunCoup web site (http://funcoup.sbc.su.se).

The principle of the network enrichment analysis is shown in Figure 2. Panel A shows a schematic diagram: in order to characterize an experimentally derived AGS with relationships to an FGS, we count the number of links between members of the two sets as given by a known background network. In this figure there are 8 links. Allowing such links expands the potential biological characterization of the altered genes using rich information on gene interaction networks. Panel B shows a realistic example from the smallest AGS from the lung-cancer proteomics data: the AGS consists of 257 deregulated proteins in one lung tumor sample (diamonds). The FGS contains 10 genes involved in epithelial-mesenchymal transition (EMT), namely TWIST1, SNAI1, SNAI2, SNAI3, SIP1, CDH1, CDH2, VIM, FN1, ACTA2. In total, 88 links were found between the AGS and FGS. Note that overlaps between AGS and FGS are allowed, and in fact these overlaps tend to produce many links; in contrast, in GEA the enrichment is based on the number of such overlaps only.

To assess the level of connectivity, we generated a series of randomized networks that preserved the degree distribution of all genes. The estimated number of links expected by chance was 51.6 and the FNEA *z*-score was 4.97. In the other 15 patients with proteomics data, the z-score associated with EMT ranged from 1.81 to 9.83, so that the current sample was ranked 6 out of 16. In total, 15 z-scores out of 16 exceeded 1.96. Importantly, two of the EMT proteins, VIM and FN1, were themselves found in eight and ten sample AGSs, respectively. As a result, GEA also detected some relationship between these tumors and EMT, but only 6 of the GEA scores exceeded 1.96. Looking at Figure 2B explains the higher sensitivity of NEA: network connections lead to ACTA2, CDH1, SIP1, and other EMT genes that were themselves not found in the AGS. In addition, FN1 and VIM contributed with many links.

### Performance on known gene-sets

To demonstrate the biological validity of NEA, we analyzed gene sets altered in our lung cancer dataset versus known gene groups relevant to this disease. For each sample, we set the AGS to be the top 100 genes DE between tumor and normal tissues, so we had a total of 123 AGSs. Ding *et al.*[26] compiled a comprehensive list of 623 genes – known tumor suppressors, growth factors etc. – and screened them for somatic mutations in lung adenocarcinoma tissues. Among the 623 genes, 26 genes were declared ‘confident’ drivers in the sense that they are mutated at significantly high frequency. We performed NEA on (i) the full set of 623 genes, (ii) the set of 26 confident driver genes, and (iii) the set of 153 genes for which there were at most one mutation was detected by [26]. Another functional group was the pathway of non-small cell lung cancer from KEGG database.

Additionally, we downloaded a list of genes somatically mutated in lung cancers from the COSMIC database [27], which is potentially less biased towards previously known cancer drivers. The list of 764 genes not included in Ding *et al.*[26] was split into two parts: (i) 110 genes that mutated more than once and (ii) 654 genes reported only once. The latter can be regarded as a negative control. To get an unbiased comparison, we took 50 randomly sampled sets consisting of 110 genes from the 654 genes. The latter lists were expected to contain many spuriously reported passenger genes, so we expect fewer links to our lung cancer AGSs.

The results are summarized in Table 2. If the 623 genes from [26] were involved in lung cancer, then we should expect some enrichment in links to our AGSs. Indeed, according to MNEA, 114 out of 123 individual AGSs had nominally significant enrichment score (z-score >1.96). Hence, the original full set indeed contained many genes involved in lung cancer, irrespective of their vulnerability to mutations. For MNEA, a comparable number of individual AGSs (95 and 102) were enriched in the subset of driver genes and rarely mutated genes. However, using the top 100 genes (FNEA), we observed a higher number of significant AGSs for the driver subset compared to the full set, while the rarely mutated subset had the fewest. Thus, FNEA showed higher specificity than MNEA for distinguishing driver genes. Finally, FNEA and MNEA showed 48 and 99 AGSs that were linked to the non-small cell lung cancer pathway, respectively (KEGG05223 in the Table).

**Table 2:** Network enrichment analysis of lung cancer-related gene sets to patient-specific altered gene sets

**Functional gene set**	**FNEA (*k = *100)**	**MNEA**	**GEA (*k = *100)**	**GSEA**
Ding et al., 2008				
All (623 genes)	66	114	1	102
Drivers (26)	74	95	21	16
Mutated ≤1 (153)	39	102	2	75
COSMIC				
Detected >1 (110)	34	33	22	30
Detected once (median)^∗^ (110)	18	52	9	28.5
KEGG05223 non-small cell lung cancer (56)	48	99	2	40
Largest contrast GEA > FNEA				
KEGG00362 Benzoate degradation via hydroxylation (3)	4	27	123	31
KEGG00513 High-mannose type N-glycan biosynthesis (4)	2	5	123	24
KEGG00780 Biotin metabolism (4)	1	13	123	11
KEGG00300 Lysine biosynthesis (5)	0	6	123	34
KEGG00785 Lipoic acid metabolism (4)	0	2	123	18
Largest contrast FNEA > GEA				
GO0001666 Response to hypoxia (163)	119	114	11	105
KEGG05200 Pathways in cancer (254)	112	118	6	99
GO0001525 Angiogenesis (129)	115	113	10	118
KEGG04010 MAPK signaling pathway (286)	107	114	4	82
KEGG04020 Calcium signaling pathway (183)	99	108	5	60

KEGG05223 is non-small cell lung cancer pathway. ‘COSMIC’ genes include the lung-cancer somatic mutations in the COSMIC database, but exclude those in [26]. ∗ indicates the median from 50 randomly sampled sets.

As for the COSMIC gene sets, even the set of multiple mutations had lower numbers of significant AGSs than the above results. Also, using FNEA, we could separate those genes that mutated once from those with more than 1 mutation, again potentially distinguishing the sets of passenger and driver mutations.

In general GEA detected only a few individuals with activated cancer pathways; see Table 2. For example, only 1 of the 123 individuals had significant enrichment of 623 cancer genes of [26], and 2 in the non-small cell lung cancer pathway. GSEA showed higher overall sensitivity compared to GEA, but GSEA appeared to be less sensitive than NEA. For example, for 26 driver genes of [26] it only identified 16 significant individuals, compared to 74 and 95 for FNEA and MNEA, respectively.

Among the most activated pathways according to GEA, we saw benzoate degradation via hydroxylation, high-mannose type N-glycan biosynthesis, biotin metabolism, lysine biosynthesis and lipoic acid metabolism. However, according to the NEA scores, few individuals were activated in these pathways. Thus, many pathways not known to be activated in cancer were found to be significant in all individuals by GEA, but not by NEA. On the other hand, putative cancer-related pathways, such as response to hypoxia, pathways in cancer, angiogenesis and the MAPK signaling pathway, were activated in most individuals according to NEA, but not according to GEA.

The results indicated that NEA can detect transcriptomic activity of cancer drivers via functional relations in the network, whereas the mutated genes themselves are not known directly. The fact that both frequently mutated genes (26 genes) and rarely mutated genes (most of the other FGSs) scored high suggests that the NEA is potentially more powerful than the mutation frequency analysis [26]. Furthermore, NEA enables investigation at the level of individuals rather than on pooled observations. Indeed, a large number of cancer drivers could not be detected by Ding *et al.*[26] or other studies due to low mutation frequency observed in the samples.

**Figure 3:**
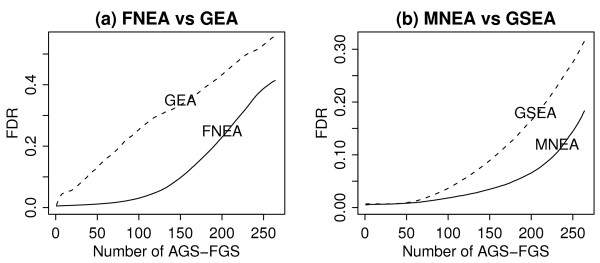
**Average of estimated FDRs versus the number of AGS-FGS pairs that are declared significant.(a)** FNEA (solid) versus GEA (dashed) **(b)** MNEA (solid) and GSEA (dashed). The average values were calculated over 123 individuals.

We further compared the procedures in terms of the false discovery rates (FDR): a better procedure should produce smaller FDR. Thus we analysed 123 individual AGSs with the 264 FGSs as described previously. Figure 3a and 3b show the average FDR curves obtained from individual AGSs. Since FNEA and GEA used a fixed number of top *k* genes, while MNEA and GSEA used the largest deviation over *k*, separate comparisons were made to compare like with like. Figure 3a indicates that for the top ranking AGS-FGS pairs, FNEA had lower FDR than GEA. Likewise, Figure 3b shows that MNEA had lower FDR than GSEA.

We also compared z-score distributions from GEA to those from MNEA and FNEA (Additional file 1: Figure S1).In general, there was little correlation between GEA and NEA scores, which would be expected as they are based on different information. In most situations NEA could achieve higher ranges of scores because there are more ways a gene can interact with other genes in a pathway than simply by being an overlap. On the other hand, the correlation between FNEA and MNEA was 0.65; since FNEA is much simpler to compute than MNEA, in practice FNEA could be sufficient for capturing the network enrichment.

### Factor analysis

Once constructed, the NEA scores from each individual can be used for further analyses. For illustration we employed factor analysis [28] in order to explore the lower dimensional space that characterized molecular profiles of individual patients. The varimax rotation algorithm is used because it often provides meaningful interpretations for the factor loadings for each variable (FGS). The individual NEA scores of FGSs served as input variables in the factor analysis. In order to make the results more interpretable, the analysis was performed separately for the following three groups of pathways: (a) cancer-related pathways; (b) signaling and cancer-related pathways; and (c) metabolic pathways. These are given in Section B of the Additional file 1 Report.

First the mean and variance of FGS scores are given in Figure S2 of the Additional file 1 Report. The cancer-related pathways were neither the most enriched nor the most variable. Many other signaling KEGG pathways exceeded them in terms of these measures. The metabolic pathways seemed as variable across individuals as signaling ones. Looking at metabolic pathways most highly correlated to signaling pathways and cancer hallmarks, we found that these correlations occurred when the same enzymes were assigned to both a metabolic and a signaling pathway. For example, the RNA-specific adenosine deaminase ADAR (atrazine degradation, KEGG00710) was related to (but not a member of) the cell cycle pathway (KEGG04110) due to its molecular function. Similarly, protein phosphatases that enable biological signaling (and hence belong to multiple signaling pathways) are classified as enzymes of the inositol phosphate metabolism (KEGG00562).

#### Cancer-related pathways

We used 16 FGSs that are the known hallmarks of cancer [25] and 13 processes relevant to tumor growth. The factor loadings revealed that variability of transcription is mostly shaped by Factor 1: PDGF, IGF, and TGF-beta signaling, angiogenesis and epithelial-mesenchymal transition (EMT), Factor 2: apoptosis and ErbB (also known as HER2 or EGFR) signaling, Factor 3: p53 signaling and cell cycle. We found that EMT was significantly enriched in the virtually all tumors (FNEA *z* score ranged from 2 to 30), so the variability across patients reflects the varying degree of activation. On the other hand, p53 signaling and cell cycle were significantly (*z* > 2) activated in subsets of individuals (79 and 24 out of 123 individuals, respectively).

#### Signaling pathways

To reveal how cancer-related pathways interact with the normal signaling machinery, we studied factor structure of the combined cancer-related pathways and 55 signaling pathways from KEGG. The first factor (21.4% of total variance) combined apoptosis with Jak-STAT pathway and other immunity-related signaling (B-cell, T-cell, toll-like receptor, and other pathways). Factor 2 explained 16.2% of the total variance. Unlike the first analysis, ErbB was placed with pathways MAPK, WNT, VEGF, insulin, gonadotropin-releasing hormone, and calcium signaling. The third factor (10.0%) confirmed joint activity of PDGF, IGF, and TGF-beta signaling, angiogenesis, and EMT pathways. In addition, these were associated with tissue growth pathways: adherens junction (KEGG04520), focal adhesion (KEGG04510), regulation of actin cytoskeleton (KEGG04810), and tight junction (KEGG04530).

Thus, by combining the cancer-related processes with KEGG signaling pathways, we found that the former do contribute significantly to the factor structure of the signaling pathways. We could see how the core cancer processes cross-talk with their signaling counterparts in the tumor transcriptome level.

#### Metabolic pathways

We previously observed that the signaling and metabolic domains of the global gene interaction network are poorly connected with each other (unpublished data). In order to overview tumor functionality at the metabolic level, we did factor analysis for 119 metabolic KEGG pathways. Factor 1 (19.2% of total variance) was dominated by metabolism of retinol, steroid hormones, ascorbate/aldarate, fatty acids and several amino acids. Factor 2 (10.6%) incorporated various pathways of sugar metabolism and oxidative phosphorylation, while factor 3 (7.4%) combined metabolism of purine, pyrimidine, phosphonate/phosphinate, seleno-aminoacids, cysteine and methionine.

### Comparing the gene and protein expression data

Distinct AGSs from the transcriptomics and proteomics data allowed a systematic analysis of both similarities and differences between individual patients at molecular level. For this we compared the NEA scores of individual AGSs versus 264 FGSs for the two omics data; the plots are given in the Additional file 1: Figure S2. Both manifested higher mean scores for the five tissue growth-related pathways: adherens junction (KEGG04520), gap junction (KEGG04540), focal adhesion (KEGG04510), regulation of actin cytoskeleton (KEGG04810), and tight junction (KEGG04530). These pathways were highly enriched in the vast majority of patients. Similarly, the score distribution of metabolic pathways was quite similar between the two platforms in terms of means and variances. Intriguingly, the proteomics AGSs were strongly depleted in cancer-related FGSs, whereas in the transcription domain the cancer genesets showed the opposite pattern. We have no explanation for this observation.

## Conclusion

We have developed a network enrichment analysis that can be considered a natural extension of the gene enrichment analysis in the network context. NEA exploits the rich and growing information on the biological interaction between genes at different levels, including DNA, RNA and protein, and from different species. The statistical power of this approach is higher than GEA, because the number of potential connections between gene groups is expanded from listed genes to all their network neighbors. The analysis is robust if the gene network is sufficiently dense, so that only connections via several network links between gene groups are considered. We provided such a global gene network of functional coupling by integration of high-throughput and literature data of [21], complementing it with functional links from curated databases of pathways, gene annotations, and protein complexes.

Our NEA method by definition is not limited to any sub-networks or modules defined by the network structure. In comparison, NEA methods that analyze pre-defined network modules then perform GEA within each module separately, and ignore all non-module genes. Algorithmic solutions for identifying network modules are often controversial, and might produce multiple solutions, where each alternative is hard to validate. Furthermore, in our experience with JActiveModules many identified modules are either very small (fewer than 5 genes) or extremely large, likely representing the largest component of the network, thus creating an additional level of uncertainty in the analysis pipeline. This direction of research can be extended beyond existing gene networks, e.g. attempting to identify latent gene structures [29].

Genes, the members of FGS used in our analyses, often overlap. This problem is common to all enrichment-based methods that employ ready, third-party defined FGS. Solutions can include either resolving ambiguity by rigorous weighted scoring [30] or creating curated, non-overlapping FGS for specifically for enrichment analyses, such as hallmarks of cancer, domains of enzymatic activity, etc.

Obtaining the distribution of the network statistics under the null hypothesis, which is needed for inference, is challenging, and we believe that this has hindered network-based analyses so far. In this paper, we propose a sequential network randomization algorithm, using the hypergeometric sampling model to obtain a fast procedure for large biological networks. Chen *et al.*[23] proposed a sequential importance sampling method to approximate the distribution of the statistics related to the randomized 0-1 tables with fixed margins. In contrast to our procedure, they did not randomize the network itself, but an approximation to it as determined by the trial distribution. Hence, finding or constructing a good trial distribution becomes an issue.

We have focused on the number of direct links with equal weights as the key statistic, but some extensions of this are worth pursuing. For example, one might consider other network statistics, such as the number of indirect links, or more generally, allow different weights for the links. In addition to analyzing an AGS in relation to known gene sets, the NEA has a unique feature: it can quantify the enrichment of single genes in the AGS. As such an analysis does not require any prior gene grouping, it can be applied to all genes found in the network. In this case the characterization of the AGS is based on any available knowledge on the enriched individual genes, such as gene description, presence of a certain protein domain, etc.

Finally, in generating the randomized networks we have constrained them only to have the have a specified degree distribution. Let network assortativity be defined as the correlation between the degrees of the two nodes connected by a link. In our randomized networks a large node is more likely to be connected another large node rather than to a small one. In other words the randomized networks tend to be assortative. The network that we use has an assortativity of *r* = 0.2, indicating that high-degree nodes are more frequently connected to high degree nodes than to low-degree nodes. We observe the same trend for the human STRING network [31] (link confidence cutoff > 0.5, *r* = 0.32). This is opposite to what was found by Newman [32] (*r* = −0.15), and Maslov and Sneppen [20] (*r* < 0). However, the networks used in their studies were relatively small yeast networks and purely based on physical protein interactions. In any case, the impact of ignoring the second-order property on network-based inferences requires further investigation.

## Competing interests

The authors declare that they have no competing interests.

## Authors contributions

AA and WJL contributed equally; VL, JL and YP conceived of this project as principal investigators. VL, JG and PD carried out the microarray experiments; JL and MP carried out the protein experiments. AA, WJL and YP performed the statistical analysis. AA, WJL, MP, JL and YP wrote the paper. All authors read and approved the final manuscript.

## Supplementary Material

Additional file 1 Has three sections. Section A has two figures: In Figure S1, we compared z-scores from GEA to those from MNEA and FNEA. In Figure S2, we compared the NEA scores of individual AGSs versus 264 FGSs for the two omics data. Section B contains the list of pathways used in our lung cancer study. Their meanings and references are also given. For pedagogic purposes, in Section C, we provide a fully worked-out example of analysis of lung cancer data from Bild *et al.*’s. Click here for file
