# Supplementary material for: Network enrichment analysis: extension of gene-set enrichment analysis to gene networks
Source: BMC Bioinformatics. 2012 Sep 11;13:226. doi: 10.1186/1471-2105-13-226 (PMC3505158; doi:10.1186/1471-2105-13-226)
Supplement: Additional file 1 — Has three sections. Section A has two figures: In Figure S1, we compared z-scores from GEA to those from MNEA and FNEA. In Figure S2, we compared the NEA scores of individual AGSs versus 264 FGSs for the two omics data. Section B contains the list of pathways used in our lung cancer study. Their meanings and references are also given. For pedagogic purposes, in Section C, we provide a fully worked-out example of analysis of lung cancer data from Bild et al.’s. [file 1471-2105-13-226-S1.doc]

**Supplementary material**

Alexeyenko et al Network enrichment analysis: extension of gene set enrichment analysis to gene networks

1. **Figures**

**Figure S1**: Scatter plots of the MNEA and FNEA vs GEA scores.


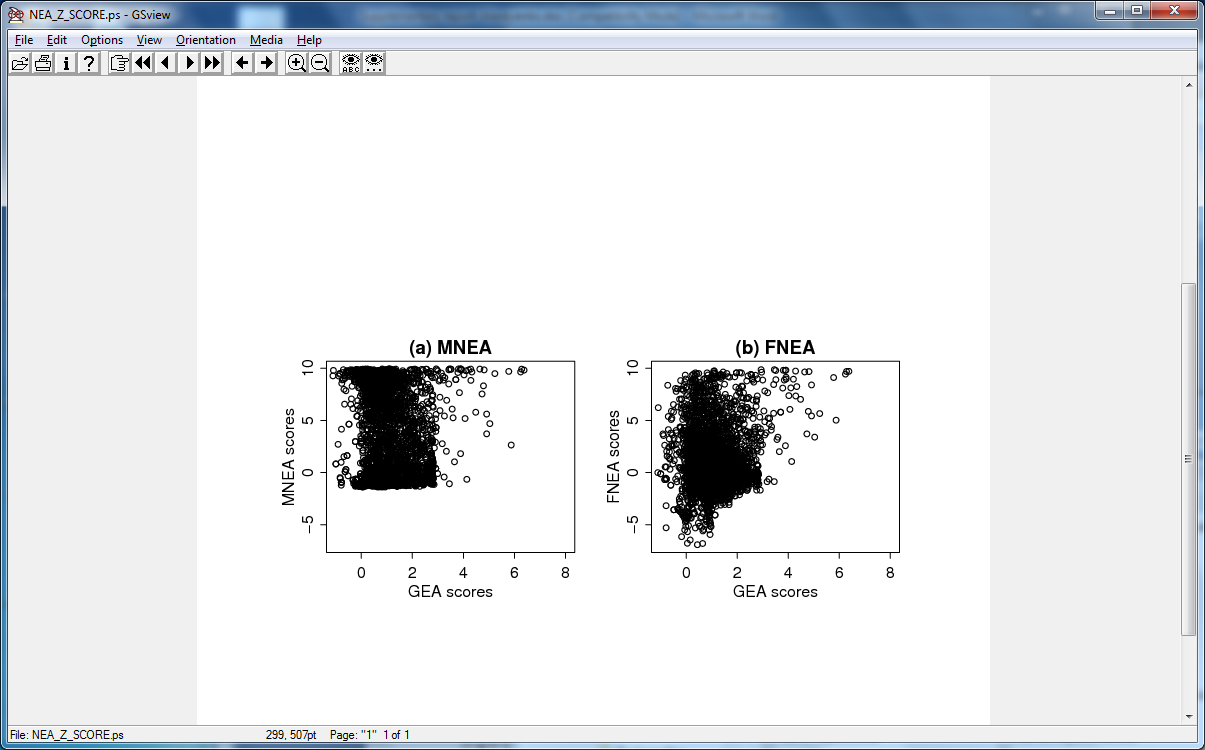


**Figure S2:** Scatterplot of the mean vs variance of the NEA z-scores; each symbol represents one sample. C= cancer-related pathways , S=signaling pathways and M=metabolic pathways. These pathways are given below.


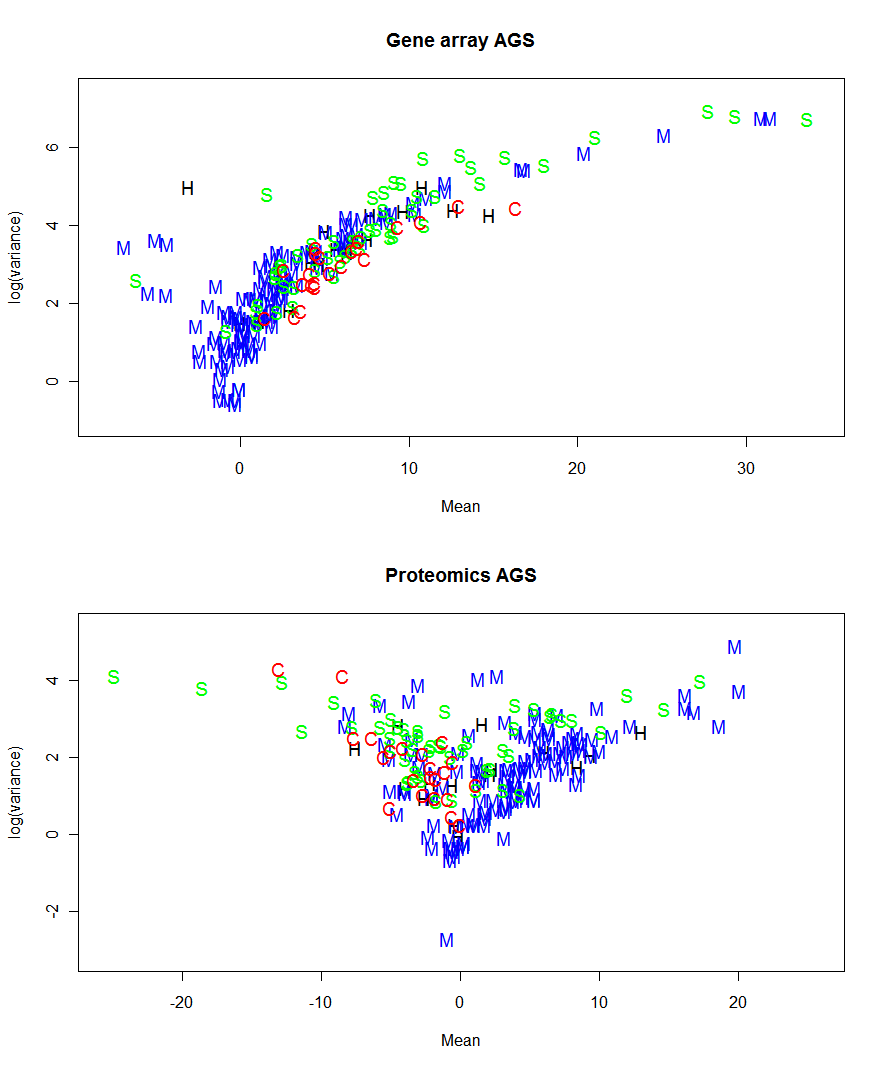


1. List of pathways

**Cancer-related patways**

1. **Hallmarks of cancer**

GO:0001525, angiogenesis

GO:0001570, vasculogenesis

GO:0001666, response to hypoxia

GO:0002347, response to tumor cell

GO:0002418, immune response to tumor cell

GO:0005154, EGFR binding

GO:0005161, PDGFR binding

GO:0005164, TNF binding

GO:0005520, IGF binding

GO:0006915, apoptosis

GO:0007179, TGFb pathway

GO:0017134, FGF binding

GO:0032570, response to progesterone stimulus

GO:0032640, TNF production

GO:0043120, TNF binding

GO:0070848, response to growth factor stimulus

**2. Additional pathways (non GO and nonKEGG)**

[1] "acidic_switch" "all623_Ding2008" "AlzheimerCore"

[4] "breast_cosmic" "EMT" "estrogen_signaling"

[7] "Jones2008pancrea_" "parp" "Parsons2008GBM_"

[10] "significant26_Ding2008" "Sjoblom2006breast_" "Sjoblom2006colorectal_"

[13] "TCGARN2008_glioblastoma"

**Explanation of these pathways:**

[1] **acidic switch**, a pathway behind tumor's ability to grow in hypoxic environment (manual curation by A. de Milito)

(16 genes) "SLC9A1" "ATP6V0C" "ATP6V0A1" "ATP6V0A2" "TCIRG1" "ATP6V0A4" "ATP6V1A" "ATP6V1C1" "ATP6V1C2" "ATP6V0D1" "ATP6V0D2" "CA9" "CA2" "TM9SF4" "SLC16A1" "SLC16A4"

[2] **all623_Ding2008**, list of candidate lung cancer drivers for somatic mutation screen (Ding et al., 2008)

The list is too long, so we refer to the Supplementary Table of Ding et al. (2008) for the

details.

[3] **AlzheimerCore**

(4 genes) "PSEN2" "PSEN1" "APOE" "APOE"

[5] **epithelial-mesenchymal transition**, a pathway behind metastatic development (manual curation by S. Souchelnytskyi)

(10 genes) "TWIST1" "SNAI1" "SNAI2" "SNAI3" "SIP1" "CDH1" "CDH2" "VIM" "FN1" "ACTA2"

[6] **estrogen_signaling**

(25 genes) "CARM1" "CYP7B1" "DDX54" "DYX1C1" "ESR1" "ESR2" "FOXA1" "FOXL2" "LEF1" "MLL2" "MMS19" "NCOA3" "NCOA6" "NKX3-1" "NRIP1" "NSD1" "PHB2" "PPARGC1B" "PSMC3IP" "RERG" "SRC" "STRN3" "STRN" "TAF10" "WIPI1"

[7] **Jones2008pancrea**_, an experimentally derived pathway of human pancreatic cancers (Jones et al., 2008)

(81 genes) "DLEC1" "CDH10" "DEPDC2" "NP_001073991.1" "ELN" "MLL3" "GLTSCR1" "ABCA7" "CTNNA2" "CYFIP1" "SLC6A15" "ZNF638"

"DLG3" "SEMA5B" "EP300" "KLHDC4" "SLC1A6" "GPR133" "MEP1A" "TBX18" "SF3B1" "TPO" "TNR" "ARID1A" "PCDH17" "PDZRN3" "MYH2" "GRIA3" "PRKCG" "DACH2" "SMARCA4" "DPP6" "PXDN" "COL5A1" "KIAA0774" "KRAS" "DOCK2" "BAI3" "Q5VY36_HUMAN" "UNC13C" "PCSK6" "TP53" "SMAD4" "ST6GAL2" "GALNT13" "ZAN" "IL2RG" "CDKN2A" "CNTN5" "PCDH15" "GUCY1A2" "CACNA2D1" "PPP1R3A" "FMN2" "NRG2" "FMNL3" "TGFBR2" "PBRM1" "ABLIM2" "EGFLAM" "DLC1" "TM7SF4" "SLITRK5" "LRFN5" "EVPL" "NP_056346.3" "K1024_HUMAN" "RASSF6" "SCG2" "MIZF" "LRRN3" "ADAMTS20" "LRRTM4" "KBTBD11" "NP_997288.1" "SCN5A" "F8" "OVCH1" "PCDH18" "OR10R2" "ATP10A"

[8] **parp** Poly (ADP-ribose) polymerase (PARP) is a family of proteins involved in a number of cellular processes involving mainly DNA repair and programmed cell death. See http://parplink.u-strasbg.fr/index.html

(20 genes) "PARP10" "PARP11" "PARP12" "PARP14" "PARP15" "PARP16" "PARP1" "PARP2" "PARP3" "PARP4" "PARP5a" "PARP5b" "PARP6" "PARP7" "PARP8" "PARP9" "TIPARP" "TNKS2" "TNKS" "VPARP"

[9] **Parsons2008GBM**_, an experimentally derived pathway of glioblastoma multiforme (Parsons et al., 2008)

(42 genes) "LRRC7" "NGEF" "LRP2" "RBM27" "GML" "PIK3CA" "TRPV5" "MYO1B" "KIAA0774" "CDK4" "KIAA0133" "IMP4" "IDH1" "RB1" "TP53" "ZNF687" "M3KL4_HUMAN" "SKP2" "PIK3R1" "PHIP" "C6orf170" “EGFR" "CDKN2A" "ASTN1" "IRX6" "LMX1A" "SERPINA12" "COL3A1" "SCN9A" "FRMPD4" "PKHD1" "PTENP1" "ARNT2" "ZNF497" "DSG4" "Q8NDH2_HUMAN" "C21orf29" "OR2L13" "HLA-DRB9" "CACNA1H" "NF1" "GRM3"

[10] **significant26_Ding2008**, list of confident lung cancer drivers after somatic mutation screen (Ding et al., 2008)

(26 genes) "TP53" "KRAS" "STK11" "EGFR" "LRP1B" "NF1" "ATM" "APC" "EPHA3" "PTPRD" "CDKN2A" "ERBB4" "KDR" "FGFR4" "NTRK1" "RB1" "NTRK3" "EPHA5" "PDGFRA" "GNAS" "LTK" "INHBA" "PAK3" "ZMYND10" "NRAS" "SLC38A3"

[11] **Sjoblom2006breast**_, an experimentally derived pathway of breast cancer (Sjoblom et al., 2006)

(122 genes) "DNAH9" "BRCA1" "ZFP64" "MRE11A" "SERPINB1" "CDH10" "SYNE2" "COL11A1" "CYB5R4" "HDAC4" "NUP133" "ZFYVE26" "CENTB1" "NLE1" "RASAL2" "RAP1GAP" "CIC" "KEAP1" "ATP8B1" "COL19A1" "SEMA5B" "RFX2" "NRCAM" "GGA1" "THOC5" "MTMR3" "SIX4" "CDH20" "ICAM5" "GRIN2D" "HOXA3" "PLEKHA8" "CUBN" "MYH1" "GAB1" "TECTA" "NP_115981.1" "GLI1" "PRPF4B" "PCDHB15" "RASGRF2" "DBN1" "COL7A1" "RRP9" "OTOF" "APC2" "SLC9A2" "HDLBP" "CHD5"

"ACADM" "BCL11A" "LRRFIP1" "CFP" "KTN1" "NUP214" "MACF1"

"MYOD1" "STARD8" "PKDREJ" "KIAA0427" "CNTN6" "HNF1A" "CENTG1"

"ABCB10" "SP110" "ARHGEF4" "FLNB" "GPNMB" "GALNT5" "SORL1"

"INHBE" "SBNO1" "CYP1A1" "TP53" "SLC6A3" "MAP3K6" "FCRL5"

"RGL1" "ITGA9" "PRPS1L1" "GSN" "ADAM12" "DIP2C" "PTPN14"

"KIAA1632" "OBSCN" "XDH" "CNNM4" "AMFR" "QSK_HUMAN" "DNASE1L3" "ZNF668" "ABCA3" "XIRP1" "SCNN1B" "THBS3" "ASL" "DPH4" "ZNF318" "RAPH1" "PPM1E" "MAMDC4" "ALS2CL" "DDX10" "NP_872431.2" "EXOC3L" "NLRP8" "EHMT1" "BGN" "C1orf64" "TMPRSS6" "C14orf155" "ZNF569" "SULF2" "KPNA5" "ABCB8" "VEPH1" "SPTAN1" "LRBA" "NCOA6" "EGFL6" "MAGEE1"

>

[12] **Sjoblom2006colorectal**_, an experimentally derived pathway of colorectal cancer (Sjoblom et al., 2006)

(64 genes) "EPHA3" "MLL3" "PTPRU" "MAP2" "RUNX1T1" "LRP2" "MMP2" "GNAS" "P2RX7" "EPHB6" "C10orf137" "FBXW7" "EYA4" "SLC29A1" "TBX22" "SFRS6" "ERGIC3" "C6orf98" "EXOC4" "LGR6" "KIAA1409" "CHL1" "APC" "LMO7" "UQCRC2" "ADAMTS18" "GALNS" "TP53" "SMAD4" "CNTN4" "HAPLN1" "PHIP" "UHRF2" "TCF7L2" "GUCY1A2" "PTPRD" "OBSCN" "ADAMTSL3" "CD109" "BRPF1" "PKNOX1" "TGFBR2" "CSMD3" "ABCA1" "ADAMTS15" "SCN3B" "SMAD3" "ADAM29" "PKHD1" "CD248" "P2RY14" "SMAD2" "MKRN3" "NP_848620.1" "PRKD1" "HIST1H1B" "KCNQ5" "C15orf2" "KRT73" "EVL" "NF1" "ACSL5" "ZNF442" "CTL4_HUMAN"

[13] **TCGARN2008gliobl_**, an experimentally derived core pathway of human glioblastoma (The Cancer Genome Atlas Research Network, 2008)

(29 genes) "CSDE1" "PIK3CB" "AKT2" "CDK6" "PIK3CG" "MET" "AKT3" "FOXO3" "CCND2" "PIK3CA" "CDKN2C" "KRAS" "PDGFRA" "CDK4" "MDM2" "SPRY2" "RB1" "TP53" "ERBB2" "AKT1" "PIK3R1" "EGFR" "CDKN2B" "CDKN2A" "FOXO1" "PTENP1" "HRAS" "NF1" "MDM4"

references:

- Jones S, Zhang X, Parsons DW, Lin JC, Leary RJ, Angenendt P, Mankoo P, Carter H, Kamiyama H, Jimeno A, Hong SM, Fu B, Lin MT, Calhoun ES, Kamiyama M, Walter K, Nikolskaya T, Nikolsky Y, Hartigan J, Smith DR, Hidalgo M, Leach SD, Klein AP, Jaffee EM, Goggins M, Maitra A, Iacobuzio-Donahue C, Eshleman JR, Kern SE, Hruban RH, Karchin R, Papadopoulos N, Parmigiani G, Vogelstein B, Velculescu VE, Kinzler KW. Core signaling pathways in human pancreatic cancers revealed by global genomic analyses. Science. 2008 Sep 26;321(5897):1801-6. Epub 2008 Sep 4.

- Parsons DW, Jones S, Zhang X, Lin JC, Leary RJ, Angenendt P, Mankoo P, Carter H, Siu IM, Gallia GL, Olivi A, McLendon R, Rasheed BA, Keir S, Nikolskaya T, Nikolsky Y, Busam DA, Tekleab H, Diaz LA Jr, Hartigan J, Smith DR, Strausberg RL, Marie SK, Shinjo SM, Yan H, Riggins GJ, Bigner DD, Karchin R, Papadopoulos N, Parmigiani G, Vogelstein B, Velculescu VE, Kinzler KW. An integrated genomic analysis of human glioblastoma multiforme. Science. 2008 Sep 26;321(5897):1807-12. Epub 2008 Sep 4.

- Sjoblom T, Jones S, Wood LD, Parsons DW, Lin J, Barber TD, Mandelker D, Leary RJ, Ptak J, Silliman N, Szabo S, Buckhaults P, Farrell C, Meeh P, Markowitz SD, Willis J, Dawson D, Willson JK, Gazdar AF, Hartigan J, Wu L, Liu C, Parmigiani G, Park BH, Bachman KE, Papadopoulos N, Vogelstein B, Kinzler KW, Velculescu VE. The consensus coding sequences of human breast and colorectal cancers. Science. 2006 Oct 13;314(5797):268-74. Epub 2006 Sep 7.

- Cancer Genome Atlas Research Network. Comprehensive genomic characterization defines human glioblastoma genes and core pathways. Nature. 2008 Oct 23;455(7216):1061-8. Epub 2008 Sep 4.

**Signaling pathways**

[1] "kegg_04010_mapk_signaling_pathway"

[2] "kegg_04020_calcium_signaling_pathway"

[3] "kegg_04060_cytokine-cytokine_receptor_interaction"

[4] "kegg_04062_chemokine_signaling_pathway"

[5] "kegg_04080_neuroactive_ligand-receptor_interaction"

[6] "kegg_04120_ubiquitin_mediated_proteolysis"

[7] "kegg_04130_snare_interactions_in_vesicular_transport"

[8] "kegg_04140_regulation_of_autophagy"

[9] "kegg_04142_lysosome"

[10] "kegg_04144_endocytosis"

[11] "kegg_04150_mtor_signaling_pathway"

[12] "kegg_04210_apoptosis"

[13] "kegg_04310_wnt_signaling_pathway"

[14] "kegg_04320_dorso-ventral_axis_formation"

[15] "kegg_04330_notch_signaling_pathway"

[16] "kegg_04340_hedgehog_signaling_pathway"

[17] "kegg_04360_axon_guidance"

[18] "kegg_04370_vegf_signaling_pathway"

[19] "kegg_04510_focal_adhesion"

[20] "kegg_04512_ecm-receptor_interaction"

[21] "kegg_04514_cell_adhesion_molecules_(cams)"

[22] "kegg_04520_adherens_junction"

[23] "kegg_04530_tight_junction"

[24] "kegg_04540_gap_junction"

[25] "kegg_04612_antigen_processing_and_presentation"

[26] "kegg_04620_toll-like_receptor_signaling_pathway"

[27] "kegg_04621_nod-like_receptor_signaling_pathway"

[28] "kegg_04622_rig-i-like_receptor_signaling_pathway"

[29] "kegg_04623_cytosolic_dna-sensing_pathway"

[30] "kegg_04630_jak-stat_signaling_pathway"

[31] "kegg_04640_hematopoietic_cell_lineage"

[32] "kegg_04650_natural_killer_cell_mediated_cytotoxicity"

[33] "kegg_04660_t_cell_receptor_signaling_pathway"

[34] "kegg_04662_b_cell_receptor_signaling_pathway"

[35] "kegg_04664_fc_epsilon_ri_signaling_pathway"

[36] "kegg_04666_fc_gamma_r-mediated_phagocytosis"

[37] "kegg_04670_leukocyte_transendothelial_migration"

[38] "kegg_04672_intestinal_immune_network_for_iga_production"

[39] "kegg_04710_circadian_rhythm_-_mammal"

[40] "kegg_04720_long-term_potentiation"

[41] "kegg_04722_neurotrophin_signaling_pathway"

[42] "kegg_04730_long-term_depression"

[43] "kegg_04740_olfactory_transduction"

[44] "kegg_04742_taste_transduction"

[45] "kegg_04744_phototransduction"

[46] "kegg_04810_regulation_of_actin_cytoskeleton"

[47] "kegg_04910_insulin_signaling_pathway"

[48] "kegg_04912_gnrh_signaling_pathway"

[49] "kegg_04914_progesterone-mediated_oocyte_maturation"

[50] "kegg_04916_melanogenesis"

[51] "kegg_04920_adipocytokine_signaling_pathway"

**Metabolic pathways**

[1] "kegg_00010_glycolysis_/_gluconeogenesis"

[2] "kegg_00020_citrate_cycle_(tca_cycle)"

[3] "kegg_00030_pentose_phosphate_pathway"

[4] "kegg_00040_pentose_and_glucuronate_interconversions"

[5] "kegg_00051_fructose_and_mannose_metabolism"

[6] "kegg_00052_galactose_metabolism"

[7] "kegg_00053_ascorbate_and_aldarate_metabolism"

[8] "kegg_00061_fatty_acid_biosynthesis"

[9] "kegg_00062_fatty_acid_elongation_in_mitochondria"

[10] "kegg_00071_fatty_acid_metabolism"

[11] "kegg_00072_synthesis_and_degradation_of_ketone_bodies"

[12] "kegg_00100_steroid_biosynthesis"

[13] "kegg_00120_primary_bile_acid_biosynthesis"

[14] "kegg_00130_ubiquinone_and_other_terpenoid-quinone_biosynthesis"

[15] "kegg_00140_steroid_hormone_biosynthesis"

[16] "kegg_00190_oxidative_phosphorylation"

[17] "kegg_00230_purine_metabolism"

[18] "kegg_00232_caffeine_metabolism"

[19] "kegg_00240_pyrimidine_metabolism"

[20] "kegg_00250_alanine__aspartate_and_glutamate_metabolism"

[21] "kegg_00253_tetracycline_biosynthesis"

[22] "kegg_00260_glycine__serine_and_threonine_metabolism"

[23] "kegg_00270_cysteine_and_methionine_metabolism"

[24] "kegg_00280_valine__leucine_and_isoleucine_degradation"

[25] "kegg_00281_geraniol_degradation"

[26] "kegg_00290_valine__leucine_and_isoleucine_biosynthesis"

[27] "kegg_00300_lysine_biosynthesis"

[28] "kegg_00310_lysine_degradation"

[29] "kegg_00330_arginine_and_proline_metabolism"

[30] "kegg_00340_histidine_metabolism"

[31] "kegg_00350_tyrosine_metabolism"

[32] "kegg_00351_1_1_1-trichloro-2_2-bis(4-chlorophenyl)ethane_(ddt)_degradation"

[33] "kegg_00360_phenylalanine_metabolism"

[34] "kegg_00361_gamma-hexachlorocyclohexane_degradation"

[35] "kegg_00362_benzoate_degradation_via_hydroxylation"

[36] "kegg_00363_bisphenol_a_degradation"

[37] "kegg_00380_tryptophan_metabolism"

[38] "kegg_00400_phenylalanine__tyrosine_and_tryptophan_biosynthesis"

[39] "kegg_00401_novobiocin_biosynthesis"

[40] "kegg_00410_beta-alanine_metabolism"

[41] "kegg_00430_taurine_and_hypotaurine_metabolism"

[42] "kegg_00440_phosphonate_and_phosphinate_metabolism"

[43] "kegg_00450_selenoamino_acid_metabolism"

[44] "kegg_00460_cyanoamino_acid_metabolism"

[45] "kegg_00471_d-glutamine_and_d-glutamate_metabolism"

[46] "kegg_00472_d-arginine_and_d-ornithine_metabolism"

[47] "kegg_00480_glutathione_metabolism"

[48] "kegg_00500_starch_and_sucrose_metabolism"

[49] "kegg_00510_n-glycan_biosynthesis"

[50] "kegg_00511_other_glycan_degradation"

[51] "kegg_00512_o-glycan_biosynthesis"

[52] "kegg_00513_high-mannose_type_n-glycan_biosynthesis"

[53] "kegg_00520_amino_sugar_and_nucleotide_sugar_metabolism"

[54] "kegg_00523_polyketide_sugar_unit_biosynthesis"

[55] "kegg_00531_glycosaminoglycan_degradation"

[56] "kegg_00532_glycosaminoglycan_biosynthesis_-_chondroitin_sulfate"

[57] "kegg_00533_glycosaminoglycan_biosynthesis_-_keratan_sulfate"

[58] "kegg_00534_glycosaminoglycan_biosynthesis_-_heparan_sulfate"

[59] "kegg_00550_peptidoglycan_biosynthesis"

[60] "kegg_00561_glycerolipid_metabolism"

[61] "kegg_00562_inositol_phosphate_metabolism"

[62] "kegg_00563_glycosylphosphatidylinositol(gpi)-anchor_biosynthesis"

[63] "kegg_00564_glycerophospholipid_metabolism"

[64] "kegg_00565_ether_lipid_metabolism"

[65] "kegg_00590_arachidonic_acid_metabolism"

[66] "kegg_00591_linoleic_acid_metabolism"

[67] "kegg_00592_alpha-linolenic_acid_metabolism"

[68] "kegg_00600_sphingolipid_metabolism"

[69] "kegg_00601_glycosphingolipid_biosynthesis_-_lacto_and_neolacto_series"

[70] "kegg_00603_glycosphingolipid_biosynthesis_-_globo_series"

[71] "kegg_00604_glycosphingolipid_biosynthesis_-_ganglio_series"

[72] "kegg_00620_pyruvate_metabolism"

[73] "kegg_00623_2_4-dichlorobenzoate_degradation"

[74] "kegg_00624_1-_and_2-methylnaphthalene_degradation"

[75] "kegg_00625_tetrachloroethene_degradation"

[76] "kegg_00626_naphthalene_and_anthracene_degradation"

[77] "kegg_00630_glyoxylate_and_dicarboxylate_metabolism"

[78] "kegg_00631_1_2-dichloroethane_degradation"

[79] "kegg_00632_benzoate_degradation_via_coa_ligation"

[80] "kegg_00633_trinitrotoluene_degradation"

[81] "kegg_00640_propanoate_metabolism"

[82] "kegg_00641_3-chloroacrylic_acid_degradation"

[83] "kegg_00642_ethylbenzene_degradation"

[84] "kegg_00643_styrene_degradation"

[85] "kegg_00650_butanoate_metabolism"

[86] "kegg_00660_c5-branched_dibasic_acid_metabolism"

[87] "kegg_00670_one_carbon_pool_by_folate"

[88] "kegg_00680_methane_metabolism"

[89] "kegg_00710_carbon_fixation_in_photosynthetic_organisms"

[90] "kegg_00720_reductive_carboxylate_cycle_(co2_fixation)"

[91] "kegg_00730_thiamine_metabolism"

[92] "kegg_00740_riboflavin_metabolism"

[93] "kegg_00750_vitamin_b6_metabolism"

[94] "kegg_00760_nicotinate_and_nicotinamide_metabolism"

[95] "kegg_00770_pantothenate_and_coa_biosynthesis"

[96] "kegg_00780_biotin_metabolism"

[97] "kegg_00785_lipoic_acid_metabolism"

[98] "kegg_00790_folate_biosynthesis"

[99] "kegg_00791_atrazine_degradation"

[100] "kegg_00830_retinol_metabolism"

[101] "kegg_00860_porphyrin_and_chlorophyll_metabolism"

[102] "kegg_00900_terpenoid_backbone_biosynthesis"

[103] "kegg_00901_indole_alkaloid_biosynthesis"

[104] "kegg_00902_monoterpenoid_biosynthesis"

[105] "kegg_00903_limonene_and_pinene_degradation"

[106] "kegg_00908_zeatin_biosynthesis"

[107] "kegg_00910_nitrogen_metabolism"

[108] "kegg_00920_sulfur_metabolism"

[109] "kegg_00930_caprolactam_degradation"

[110] "kegg_00940_phenylpropanoid_biosynthesis"

[111] "kegg_00944_flavone_and_flavonol_biosynthesis"

[112] "kegg_00950_isoquinoline_alkaloid_biosynthesis"

[113] "kegg_00960_tropane__piperidine_and_pyridine_alkaloid_biosynthesis"

[114] "kegg_00970_aminoacyl-trna_biosynthesis"

[115] "kegg_00980_metabolism_of_xenobiotics_by_cytochrome_p450"

[116] "kegg_00982_drug_metabolism_-_cytochrome_p450"

[117] "kegg_00983_drug_metabolism_-_other_enzymes"

[118] "kegg_01040_biosynthesis_of_unsaturated_fatty_acids"

1. A fully worked-out example using Bild’s et al (2006)’s lung cancer data (all the necessary files are given in www.meb.ki.se/~yudpaw)

## Bild dataset: Bild et al. (2006)

## Reference: Oncogenic pathway signatures in human cancers as a guide to targeted theraphies.

## Nature, 439, pp353-357

## Their research purpose was to identify gene expression signatures of human cancers that reflect

## the activity of a given pathway.

## The gene expression dataset for lung cancer consists of 53 squamous cell carcinomas (SCC) and 58 ## adenocarcinomas (AC).

## In this example, we will apply the NEA method to a list of differentially expressed genes of 53 SCC ## versus 58 AC.

## The expression dataset was obtained using Human U133 2.0 plus arrays (Affymetrix) containing

## 56475 gene probes.

## It is available at http://data.cgt.duke.edu/oncogene.php.

## For the downstream analysis, we normalized the dataset for each patient to have zero mean after ## taking logarithm.

load("BildExample.RData") ### This includes datasets and some basic functions.

load("mergedNet.RData") ### This includes the information of the network connectivity .

load("permuted-network-100.RData") ### This is needed to reduce the computational time.

require(nea) ### The current version of the package is 1.1.1.

pid<-Clindat.bild[,"Patid"]

GEset = bild.GSE3141.GE[,pid];Patients<-pid

outcomeLabels <- Clindat.bild[pid,"Relapse"]; names(outcomeLabels)<-Patients;

tstat.bild<-tstatistics2(xdat=GEset,grp=outcomeLabels)$tstat

pval.bild<-2*(1-pt(abs(tstat.bild),df=ncol(GEset)-2))

## To see the distribution of p-values

hist(pval.bild)

### rownames(GEset) ## We need to transform the rownames to gene symbol for nea.

### chip: Human U133 2.0 plus arrays (Affymetrix)

library("hgu133plus2.db") ### This DB is downloaded in 03-05-2012.

x <- hgu133plus2SYMBOL

affyid<-rownames(GEset)

nid<-length(affyid)

genesym<-rep(0,nid)

for (i in 1:nid){

genesym[i]<-x[[affyid[i]]]

genesym[i]<-toupper(genesym[i])

if ((i %% 1000)==0) print(i)

}

sum(is.na(genesym)) ## 13427

### FNEA : AGS is the top 100 genes having smallest p-values.

top100<-order(pval.bild)[1:100]

AGStemp<-genesym[top100]

miss<-which(is.na(AGStemp)==1) ### We omit NAs

AGS_FNEA<-AGStemp[-miss] ### length=76

## Our target FGS is KEGG05223

### FNEA

res.FNEA<-nea(ags=unique(AGS_FNEA),fgs=KEGG05223,fgslib=NULL,network=mnet,pnet=NET,nperm=50,stat="F",seed=1234)

##zscore=10.52125

### MNEA : AGS consist of the top 1000 genes having smallest p-values.

top1000<-order(pval.bild)[1:1000]

AGStemp<-genesym[top1000]

miss<-which(is.na(AGStemp)==1) ### We omit NAs

AGS_MNEA<-AGStemp[-miss] ### length=742

res.MNEA<-nea(ags=unique(AGS_MNEA),fgs=KEGG05223,fgslib=NULL,network=mnet,pnet=NET,nperm=50,stat="M",seed=1234)

##zscore=8.749797
